# A Web-based computer-tailored game to reduce binge drinking among 16 to 18 year old Dutch adolescents: development and study protocol

**DOI:** 10.1186/1471-2458-14-1054

**Published:** 2014-10-09

**Authors:** Astrid Jander, Rik Crutzen, Liesbeth Mercken, Hein de Vries

**Affiliations:** Department of Health Promotion, Maastricht University, School for Public Health and Primary Care CAPHRI, Maastricht, The Netherlands

**Keywords:** Alcohol use, Binge drinking, Adolescents, Web-based interventions, Computer-tailoring, e-Health, I-Change model

## Abstract

**Background:**

In the Netherlands, excessive alcohol use (e.g., binge drinking) is prevalent among adolescents. Alcohol use in general and binge drinking in particular comes with various immediate and long term health risks. Thus, reducing binge drinking among this target group is very important. This article describes a two-arm Cluster Randomized Controlled Trial (CRCT) of an intervention aimed at reducing binge drinking in this target group.

**Methods:**

The intervention is a Web-based, computer-tailored game in which adolescents receive personalized feedback on their drinking behavior aimed at changing motivational determinants related to this behavior. The development of the game is grounded in the I-Change Model. A CRTC is conducted to test the effectiveness of the game. Adolescents are recruited through schools, and schools are randomized into the experimental condition and the control condition. The experimental condition fills in a baseline questionnaire assessing demographic variables, motivational determinants of behavior (attitude, social influences, self-efficacy, intention) and alcohol use. They are also asked to invite their parents to take part in a short parental component that focusses on setting rules and communicating about alcohol. After completing the baseline questionnaire, the experimental condition continues playing the first of three game scenarios. The primary follow-up measurement takes place after four months and a second follow-up after eight months. The control condition only fills in the baseline, four and eight month follow-up measurement and then receives access to the game (i.e., a waiting list control condition). The effectiveness of the intervention to reduce binge drinking in the previous 30 days and alcohol use in the last week will be assessed. Furthermore, intention to drink and binge drink are assessed. Besides main effects, potential subgroup differences pertaining to gender, age, and educational background are explored.

**Discussion:**

The study described in this article gives insight into the effectiveness of a possible solution for a prominent public health issue in the Netherlands, which is binge drinking among 16 to 18 year old adolescents.

**Trial registration:**

Dutch Trial Register (NTR4048). Trial registered on 06/26/2013.

## Background

Before 1st of January 2014, Dutch adolescents were allowed to buy low-strength alcoholic beverages (with alcohol percentage by volume ≤15%) when they turned 16. This age limit has just recently been increased to 18 years for any alcoholic beverage [1]. Alcohol use among Dutch adolescents is very high compared to other European countries as on average 57.4% of the 16 year old and 61.9% of the 17 to 18 year old adolescents engaged in binge drinking (drinking 4/5 or more glasses of alcohol in one occasion for girls/boys) at least once in the previous month [2]. Alcohol use, particularly during adolescence, has various consequences for the adolescent’s physical, social- and intellectual health, such as physical fighting, being injured [3], experiencing sexual assaults, dating violence, unwanted pregnancies, smoking and using illicit drugs [4–6], decreased school performance, brain damage and cognitive deficits [4, 7]. To date, most studies have focused on adolescents younger than 16 years [8–12] or young adults older than 18 [13–15], and not on the target group in our study, which are 16 to 18 year old adolescents.

The Internet has become a medium many people worldwide use to search for all kinds of health related and non-health related information. A promising way to change health related behaviors using the Internet is by means of Web-based computer tailoring (CT), where participants receive highly personalized feedback on their behavior or related socio-cognitive determinants (e.g., attitude, self-efficacy) [16]. Web-based interventions have the possibility to reach large numbers of people, and have repeatedly proven to be effective in changing various health behaviors and their determinants [17–19]. However, these interventions also suffer from a major drawback which is drop-out of intervention participants during the intervention [20–22]. Further, effect sizes are often small to medium [17]. In an attempt to maximize effectiveness of an intervention to reduce alcohol use and binge drinking in Dutch 16–18 year old adolescents with the shortcomings of CT interventions in our mind we started with conducting three different studies as formative research for the development of our intervention: a focus group study, an expert Delphi study and a questionnaire study. In the following paragraphs we briefly describe these studies’ aims and the results as background information for the development of the intervention.

Focus group interviews were conducted with 16 to 18 year old adolescents, and with parents of 16 to 18 year old adolescents. The aim of the interviews was to obtain insight into the determinants of alcohol use and particularly binge drinking among adolescents that were, at the time of this study, legally allowed to buy low-strength alcoholic beverages. Prominent findings from this study [23] were that the most important drinking situations were at a party, in a bar and being together with friends. Although adolescents did not feel direct pressure to drink, they reported that when alcohol is available and friends are around, then there is a certain pressure to drink. Furthermore, we found a discrepancy between what adolescents thought about acceptable amounts of alcohol that their parents would approve and what parents said. Adolescents thought that their parents are just fine with their alcohol consumption as long as they do not get drunk or show visible signs of drunkenness (e.g. vomiting). Parents, on the other hand, said that the acceptable limits of alcohol that their child is allowed to drink were around two glasses of alcohol. This discrepancy reveals that the communication concerning alcohol use between parents and their child is unclear. Regarding the interviews with the parents, the most important findings were that parents were aware of the negative consequences of alcohol use in adolescents, but in general stopped setting rules about alcohol use when their children turned 16. The mostly given reasons for stopping setting rules were that parents thought, prohibiting, or limiting alcohol use would be useless, because alcohol was easily available in grocery stores, the child could buy alcohol without permission of the parent, and that the children are not always present and thus not controllable to the parent.

Subsequently, in a three round expert Delphi study (Jander et al., submitted for publication), we asked experts in the field of alcohol use in adolescents, what strategies could be used to successfully decrease alcohol use in adolescents in a Web-based computer-tailored intervention. We were interested in two aspects: strategies in interventions that targeted adolescents directly, and interventions that were targeted at parents with the aim to reduce alcohol use in adolescents. Main conclusions from this study were: adolescents should be given the opportunity to try out different reactions and observe the consequences of these reactions; they should be provided with refusal skills; and they should be given opportunities to cope with negative emotions in another way than drinking. For the parents the main conclusions were: parents should be advised to have clear and consistent rules; to communicate with the adolescent about alcohol use; and finally to monitor the friends and whereabouts of the adolescent. Being responsive and interested as a parent was another important feature that the experts pointed out. In addition, we asked the experts to come up with strategies to decrease drop-out of Web-based CT interventions. The experts mentioned strategies like using incentives and reminders to reduce drop-out of adolescents, but they also made suggestions about design and content of the intervention, such as the use of highly relevant material and personalized feedback, providing little text and much interaction, using an attractive design, and language that relates to the adolescent.

Finally, based on the results of the focus groups, previous research and the I-Change Model we developed a questionnaire. The I-Change Model integrates insights from various social cognitive, social-ecological and self-regulation theories [24, 25]. The questionnaire was used to identify the most important determinants for alcohol use and binge drinking in adolescents (not published), but also to investigate the influence of rules and communication about alcohol on the child’s alcohol use. Results from this study indicated that stricter rules were associated with less alcohol consumption and less binge drinking occasions (Jander et al., submitted for publication). More importantly, the results showed that the protective influence of rules on drinking behavior of the child was the same in situations where the parents were present, as in situations where the parents were absent, implying that the concern of parents that they cannot influence drinking behavior because they cannot control the child all the time may be unnecessary.

The goal of the current article is to give a detailed description of the development and components of the intervention, as well as a protocol for the two-arm Cluster Randomized Controlled Trial (CRCT) to test the intervention’s effectiveness. Based on the focus group study [23] and the Delphi study (Jander, et al. submitted for publication) we decided to develop a Web-based game for adolescents, in which we embedded computer-tailored feedback on behavior and motivational determinants. A game might be an attractive tool to keep adolescents motivated [26] and offer some degree of interaction, as recommended by the experts, and thus reduce drop-out. For the parents we designed a website on which they had the opportunity to get computer-tailored feedback on communication and setting rules concerning alcohol use.

## Methods

### Development process

Several brainstorm sessions were conducted with the research team and students, health communication experts, ICT and game design students and experts to obtain insight into possibilities, what is already available and used successfully. Wishes of the target group were also identified during the focus group interviews [23]. Finally, we talked with several serious gaming companies about our ideas. After deciding on one serious gaming company to work with we started a Facebook page where we invited a convenience sample of 24 adolescents to befriend us and join the “Facebook panel” group. We presented all material that we and the gaming company developed to this panel and received feedback that we could use to improve the material. All panel members were between 16–18 years old. The panel consisted of eight boys and sixteen girls. Twelve adolescents came from pre-university education and the other twelve had a secondary vocational education background. We contacted the panel 10 times during the development process, to present them with new material, or ask them for feedback regarding certain aspects of the game (e.g. its name, screenshots and characters of the game, realistic scenario’s after drinking too much alcohol, realistic advices for adolescents that are trying to drink less in tempting situations, layout and design of the first version of the game etc.). For complete and thoroughly participation in all 10 rounds the adolescent received a gift card worth €35.

We also invited a convenience sample of parents to join a similar panel, where we presented material for the parental website. In total 14 parents participated in this panel. We asked them to visit the website and give us feedback on layout and design of the website, usability and the content. We furthermore asked them to give us feedback on an example of a tailored letter that parents in the intervention would receive after responding to a questionnaire. We finally asked them through which methods we could reach parents to invite them to the intervention. The parents also received a gift card worth €35 after completing participation.

### Theoretical model

The theoretical model underlying the computer tailoring component of this intervention was the integrated behavioral change (I-Change) model [16, 24, 25], as this model has previously successfully used in computer-tailored interventions [27, 28]. Features that distinguish the I-Change Model from traditional models such as the Theory of Planned Behavior [29] is that the model acknowledges a pre and post motivational phase in the behavior change process as well as predisposing and information factors that influence the development of cognitions and behavior. The pre motivational stage is characterized by motivational determinants (i.e. attitude, social influences and self-efficacy) and awareness factors (i.e. knowledge, risk perception, cues to action). Intention is the factor that is most proximal to behavior. When a person is ready to actually change behavior, action plans (exact plans what to do in a predefined situation to perform a certain behavior) help the individual to so. If behavior change has taken place the person is in the post motivational phase and coping plans (plans how to cope with difficult situations) are important for maintaining the behavior change [24, 30, 31]. Predisposing factors (i.e. behavioral factors, psychological factors, biological factors, social and cultural factors) and information factors (i.e. message, channel, source) also belong to the pre motivation phase.

### Study design

We conduct the study on Dutch schools of higher secondary education and lower secondary and tertiary education, because this is the most convenient place to reach adolescents. Thus, a CRTC, with one experimental and one waiting-list control condition randomized at school level, is used to test the effectiveness of the intervention to reduce binge drinking in 16 to 18 year old adolescents. Originally, the study consisted of a baseline measurement in October 2013 followed by the intervention and a six months follow-up measurement (April 2014). In the beginning of 2013 the Dutch government started a debate about rising the legal buying age for adolescents from 16 years to 18 years per 1st of January 2014. Because our target group comprises Dutch adolescents aged 16 to 18 years, we decided to adapt the original planning, in order to avoid that the adolescents in our intervention were legally allowed to buy alcoholic drinks at baseline and were no longer allowed to buy alcohol at the follow-up measurement. Deciding to delay the baseline measurement till January 2014 after the new law was implemented, had consequences for the follow-up measurement as well. As our study is school based and a six months follow-up after baseline would fall into the summer holidays of the schools, we decided to do the primary follow-up a little earlier, after four months. Because we are also interested in the effects of the intervention after a longer time period we also included a secondary eight-month follow-up after the summer holiday. Unfortunately, a part of our target group will then be graduated so we will not be able to do the second follow-up in schools, but rather have to reach the adolescents outside school.

### Sample size estimation

The sample size estimation is based on a 10% reduction in binge drinking occasions in the preceding 30 days in the experimental group compared with the control group. Since adolescents will be nested in schools a CRCT is needed. Using a conservative approach with an effect size of 0.2, an ICC of 0.02, power of .80, significance level of 0.05, and considering drop-out of 50% of adolescents at primary follow-up, we aim to include 34 schools at baseline.

### Participants

Participants of this study are adolescents aged 16 to 18 years. Participants are recruited by sending letters containing flyers with short information about the newly developed intervention using a game to reduce binge drinking in adolescents to schools of higher and lower secondary and tertiary education in the Netherlands. The schools can get more information on the intervention website, or contact the researchers directly via telephone or mail. After a couple of weeks, if the school has not responded to the letter yet, schools are called and asked if they had received the letter and if they would like to get more information about the intervention. If they register to participate in the study, they are randomly assigned to either a control or experimental condition. Schools are not blind to their condition, because the experimental schools have to schedule a total of three lessons (two lessons in January/February for the baseline questionnaire and three game scenarios and one for the follow-up measurement in May/June) for the current study, while control schools just have to schedule two lessons (one lesson in January/February for the baseline questionnaire and one for the follow-up questionnaire in May/June). Schools also have to sign and return a consent form, in which they indicate to agree to take part in a scientific study. Before the adolescents can start with the questionnaire they also have to give informed consent to participate in this scientific study, by checking a checkbox. If adolescents refuse to give consent, they are informed that without consent they cannot participate in the study.

### Ethical approval and trial registration

The study protocol was approved by the Medical Ethics Committee of Atrium Orbis Zuyd (METC number: 12-N-104) and the study was registered at the Dutch Trial Register (NTR4048) URL: http://www.trialregister.nl/trialreg/admin/rctview.asp?TC=4048.

### Intervention

**Figure 1:**
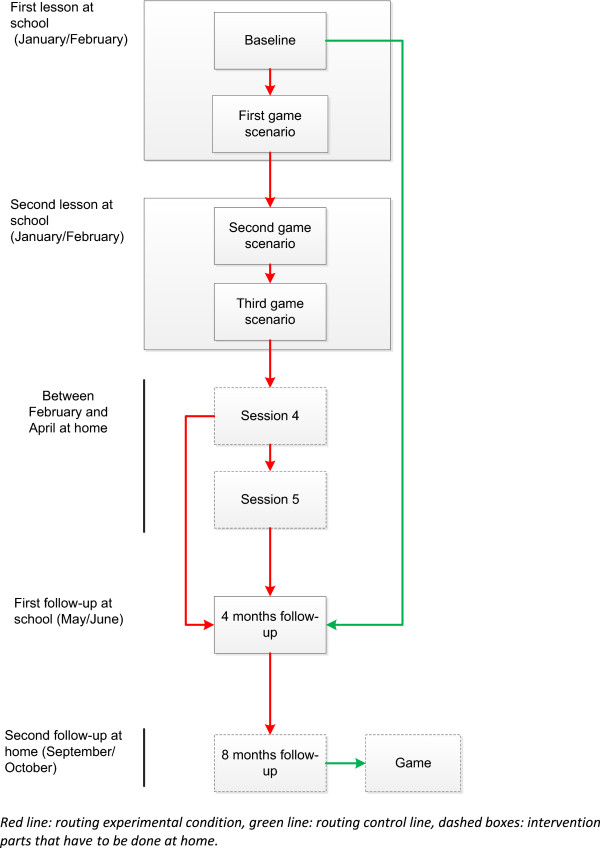
**Flowchart of the intervention.**

To enter the intervention, adolescents go to the intervention website and create an account. In the account they select their school, which will lead them into the correct routing for the control or experimental condition. The intervention consists of five sessions. A baseline questionnaire followed by three different game scenarios (i.e., sessions 1–3), a fourth session in which adolescents can accept a challenge to drink less at an upcoming drink event and finally a session to evaluate the challenge (Figure 1). The control condition fills in the baseline questionnaire only, while the experimental condition continues with the first game scenario.

### Baseline

First, adolescents have to give consent to participate in this study and then start with responding to a baseline questionnaire, assessing demographics (gender, age, educational background, family composite), alcohol use in the past week, binge drinking in the last 30 days, and situation specific alcohol use (for three situations: drinking in a bar, drinking at a party and drinking at home) motivational determinants (attitude, modeling, social norm, perceived pressure, self-efficacy, action plans), and intention to decrease alcohol use. After the baseline questionnaire the adolescents in the experimental condition immediately start with the first out of three game scenarios (Sessions 1–3).

### Game scenarios

All scenarios start the same: the adolescent wakes up in the morning after a night of partying and does not remember what happened the last night. Goal of the game is to find out what happened. This is also reflected in the title of the game “Watskeburt?!” (Dutch slang for what happened?!). There are three different drinking situations outlined in the game, one per scenario (Table 1). The order of the scenarios is also tailored so the adolescents starts with the drinking situation that he/she indicated in the baseline questionnaire that he/she drinks the most alcohol in.

**Table 1:** **Description of scenario’s**

Game scenario	Drinking location	What happened?
1	In a bar	Lost wallet and cell phone
2	At a party	Embarrassing pictures of him/her taken and put on internet
3	At a friends’ place	Fell with bike on way back home, hurt knee and lost keys

### Feedback

**Figure 2:**
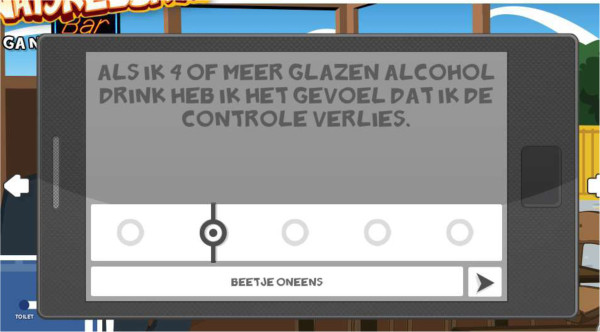
**Example of a question about attitude on the in-game cell phone.**

In every scenario, the adolescents get questions and computer-tailored feedback on an in-game cellphone (Figure 2) displayed at two moments during each scenario. The methods we use in the tailored feedback vary a little depending on the message, but usually we start with repeating the respondents answer to enhance self-monitoring, we than confirm correct assumptions with positive feedback or correct wrong assumptions with new information. We provide a personal tone in the messages and show sympathy to enhance commitment [32].

The questions and feedback of the first text message in every scenario are presented in a fixed sequence independent of the scenario. In the first text message of the first scenario the adolescent gets questions about the pros and cons of binge drinking and receives feedback on his or her overall attitude and then for every pro and con specifically. In these feedback messages the focus is on providing the participant with general and individual consequences of alcohol in order to change attitude in a more negative fashion. In the first text message of the second scenario the adolescent gets questions and feedback about social modeling of alcohol use and binge drinking. These feedback messages are provided to help the adolescent to choose the right role models, or to encourage them to seek support from friends or family who are not using much alcohol. In the first text message of the last scenario, questions and feedback about social norm and perceived pressure are provided to the adolescent. Feedback is provided with instructions how to resist pressure from friends or family to drink and provides information about perceived approval of drinking from family or friends.

**Figure 3:**
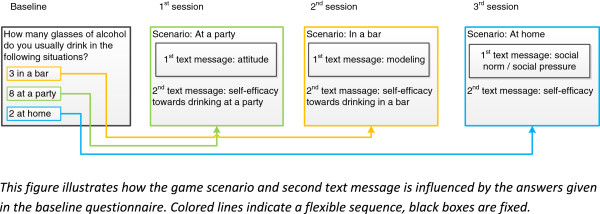
**Example of routing sessions and game scenarios.**

The content of the second text message of every scenario is about self-efficacy and action plans and are specific to the situation outlined in the scenario. This means that in the bar drinking scenario the questions in the second text message assesses self-efficacy not to binge drink in a bar, and then provides the adolescent with specific action plans how to refuse alcohol in a bar situation. In the party situation these questions are specific to self-efficacy to refuse a drink at a party etcetera (Figure 3). We chose for this sequence based on the Ø pattern [33] which describes that people shift towards behavior change through first developing a favorable attitude, experiencing positive social influences and finally developing high self-efficacy towards the behavior.

### Session 4

One week after the last game scenario the adolescents receive an e-mail inviting them back to the intervention to answer a couple of questions about their alcohol use in the previous week. They receive feedback on their drinking behavior compared with their drinking behavior at baseline and receive information whether or not they comply with the national drinking guidelines. This is done in order to raise awareness about their own drinking behavior, as awareness is an important pre motivational determinant in the I-Change Model [24]. They are then asked if they have a drinking event in the upcoming 30 days where they usually drink more than 4 (if the participant is a girl) or 5 (if the participant is a boy) glasses of alcohol. If they confirm such an event they are asked if they would like to challenge themselves to drink less than 4/5 glasses of alcohol on that event. If they accept the challenge, they have to indicate the date on which that particular event takes place and what kind of event it is (a party, a night out etc.).

After this information they are invited to make their own action plan in order to support them in their attempt not to drink more then 4/5 glasses of alcohol, or if they do not want to make their own plan, they are given a list of plans and can choose what plan they would most likely follow. Action plans are tools that are important in the action phase of behavior change [24] and can help the adolescent bridge the intention behavior gap. After deciding on a plan, they receive all the feedback from the previous three sessions to booster their memory. One day ahead of the drinking date they receive an email that prompts them about the drinking event the next day, and that they had accepted a challenge not to drink more than 4/5 glasses of alcohol. This mail was meant as a prompt to self-monitor their behavior at the drinking event.

### Session 5

One day after the drink event adolescents that accepted the challenge are invited to respond to a short questionnaire about the drink event. They indicate whether or not they achieved to drink less than 4/5 glasses of alcohol. If they indicate that they drank more they are asked for the reasons. Adolescents can indicate whether their drinking was mainly influenced by themselves, their surroundings, a combination of both or none of these. Consequently, they receive attributional feedback on the reason, providing them with information about external and internal reasons for behavior and how they can exert some influence on them. In this feedback on performance the adolescent is encouraged to continue trying to reduce the alcohol intake and to use a cue reminder (an object that helps them to remember their goal to drink less alcohol) at the next drink event. Cue reminders have been shown to help adolescents inhibit their alcohol use [34]. After that, adolescents can repeat the challenge if they wish to.

### Invitations and reminders

When participants create an account, they have to provide their e-mail address. This e-mail address is used to send participants invitations and reminders to participate. The first invitation is send to the participants of the experimental condition who have not completed the three game scenarios that they were supposed to play at school. After a week they receive a reminder. The same procedure happens for both conditions when participants do not complete the first follow-up measurement at school. Eight months after they have created an account, participants are invited to respond to the last follow-up questionnaire. If they do not respond they receive a first reminder after one week and a second reminder after two weeks.

### Parental component

Adolescents who take part in the study at school invite their parents to an additional session within the intervention. Adolescents are given the opportunity to enter the e-mail address of one of their parents in the baseline questionnaire. The parent then receives a link to the parent component the next day. If the parent is willing to participate, he/she has to give informed consent by checking a checkbox. The intervention for parents consists of a questionnaire and computer-tailored feedback. In the questionnaire we assess demographic variables (gender, land of birth, family constellation), parenting styles (involvement, psychological control, monitoring), drinking behavior, acceptable alcohol use of the child, rules concerning alcohol use, communication about alcohol use, motivational determinants (intention to talk to child and set rules, modeling, social norm, self-efficacy, action plans concerning communication and setting rules).

After responding to all questions the parent receives immediate computer-tailored feedback. First, they receive feedback what kind of parenting styles fits the information they provided and then get information about how the different parenting styles affect drinking behavior in adolescents. Following this, parents get feedback about their attitude, social influences, self-efficacy, and action plans. They further get information about how to talk with their child about alcohol and how to set appropriate rules concerning alcohol use.

After parents finish the intervention they can visit a website where they can find more general information about alcohol use and effects, and how to set rules and communicate with the child.

### Measurement instruments

The following measures are assessed among adolescents.

We measure the following demographic characteristics: gender, age, educational background (higher secondary education, lower secondary and tertiary education), religion (Catholic, Protestant, Muslim, other religion, no religion) and ethnicity (Dutch, Antilles, Belgium, German, Suriname, Moroccan, Turkish, other).

We assess *Weekly drinking behavior* with two questions. Adolescents indicate for each day of the past week if they had been drinking alcohol and, if they did, how many glasses of alcohol they had been drinking. Based on this information we calculate the total amount of alcohol they had been drinking in the past week [35]. We furthermore assess *Binge drinking* (i.e. having 4/5 or more glasses of alcohol in one occasion for a girl/boy) in the previous 30 days [36], with an open-ended question asking adolescents how many binge drinking occasions they had in the previous 30 days.

*Intention* to reduce current alcohol use is measured by two items “Are you intending to generally reduce your drinking in one occasion (e.g. in a bar, at a party etc.)” and “Are you intending to drink less than 4/5 glasses of alcohol in one occasion (e.g. in a bar, at a party etc.)”. Answers can be provided on a five-point Likert scale (1 = absolutely will not; 5 = absolutely will).

Four items measuring pros (e.g. “Binge drinking helps me having fun with friends”) and four items measuring cons (e.g. “Binge drinking makes me feel out of control”) of binge drinking are used to assess *attitude*. Participants indicate their answer on a five-point Likert scale (1 = absolutely disagree; 5 = absolutely agree). The items were derived from another study [37] using eight pros and cons. After pre-testing the questionnaire, only four important pros and cons remained in this study.

Social influences are assessed by three concepts: social modeling, social norm, and perceived pressure. *Social modeling* is assessed by asking participants how often (1 = never; 4 = very often) people in their direct environment (i.e. parents, siblings, (best) friend(s), boyfriend/girlfriend) drink alcohol and engage in binge drinking. *Social norm* is measured for each person in their direct environment (i.e. parents, siblings, (best) friend(s), boyfriend/girlfriend) by one item “My (e.g. girlfriend) thinks that … 1 = “I am not certainly allowed to binge drink” to 5 = “I am certainly allowed to binge drink”. *Perceived pressure* is assessed by “Did you ever feel pressure to drink 4/5 or more glasses of alcohol by your …?” for each person in their direct environment answered with 1 = never and 5 = always.

*Self-efficacy* is measured by ten items. Each item assesses whether participants feel able not to binge drink in a certain difficult situation (situations that would usually trigger binge drinking, e.g. “How easy or difficult is it for you to drink less than 4/5 glasses of alcohol if you are at a party?”). Participants can indicate their answer on a five-point Likert scale (1 = very difficult; 5 = very easy).

We provide participants with 21 different action plans that they could perform in order to make it easier not to binge drink in difficult situations (e.g. “Alternate alcoholic drinks with non-alcoholic drinks”). Participants indicate on a five-point Likert scale how likely it is that they will perform each action plan (1 = will I certainly not do; 5 = will I certainly do).

### Primary and secondary outcome

Primary outcome of this study is the reduction in binge drinking occasions in the previous 30 days at 4 months follow-up. The secondary outcome concerns reduction in alcohol use in the previous week and intention to reduce alcohol use and binge drinking. We will furthermore look at reduction in excessive drinking (i.e. drinking 10 or more glasses of alcohol at one occasion in the previous week) [38]. Our main focus will be the outcome measures at four months follow-up. Secondarily, we will also use the outcome measures at eight months follow-up. Additional analyses explore potential sub group differences concerning the effectiveness of the program between gender, age and high and low educated groups.

### Process evaluation

To assess level of personalization and appreciation we ask the participants after every game scenario if they thought the feedback and the game were useful, realistic and personally relevant. Answers can be provided on a four point Likert scale (e.g. 1 = very unrealistic; 4 = very realistic). They furthermore rate every advice and the game with a school grade (1 = very bad, 10 = excellent).

### Statistical analyses

General descriptive statistics will be used to describe the baseline characteristics of the participants. As the adolescents are nested in schools we will use a multilevel regression approach with three levels to assess the effects of the intervention on behavior. The first level are the repeated measures within the participants (baseline and two follow-up measurements), the second level are the pupils and the third level are the schools, where the pupils are nested. Multilevel linear regression will be used to analyze the effects of the program on week consumption and intention and multilevel logistic regression will be used to analyze the effects on binge drinking and excessive drinking. As covariates we will include condition, age, gender, educational background, religion, and the outcome variable on baseline. Moderation analyses will be performed to assess different effects for low and high educational level, age, and gender.

## Discussion

This study protocol describes a study to test the effectiveness of an intervention aimed at reducing alcohol use and specifically binge drinking in 16 to 18 year old Dutch adolescents. Reducing alcohol use at an adolescent age is of particular importance, not only because of the immediate dangerous consequences of alcohol, like getting into fights or unwanted pregnancies [3, 4], but also to reduce the risk of long term damages of the brain [7] and reducing the risk of becoming alcohol dependent later in life [39, 40]. So, reducing alcohol use at an adolescent age also reduces the risk of more long-term public health problems.

An important problem of computer-tailored interventions is that they suffer from high drop-out rates [20–22]. To minimize this, we followed the principles of social marketing [41] and conducted studies with the target group [23] and invited various experts to reflect on important issues for program development (Jander, et al. submitted for publication). This formative work revealed that the intervention should be presented in a very attractive, interactive way, hence a game. Using serious games (games with the goal to educate the gamer) to educate people about health behavior have been shown to increase motivation, knowledge, and to change health behaviors [42–45], so the idea of a game seems a suitable solution. Furthermore, we collaborated with the target group in the development of the game and intervention material (i.e. the adolescent Facebook panel and parent panel). Involving the target group in the developmental process of the intervention enabled us to build a program that took into account the wishes and preferences of the target group right from the beginning. Finally, we pilot tested the intervention at 5 schools to test the feasibility of the recruitment strategy, the design and the content of the intervention. Based on this pilot, we shortened the game and had the feedback messages shortened and rewritten by a professional writer to make them more appealing to our target group. The revised version of the intervention will be used in the trial described here.

Taken together, this study will give insights into the effectiveness of the intervention to reduce alcohol use among 16 to 18 year old adolescents and whether the development process of the game limited drop-out in the trial.
